# Recipient UvrD helicase is involved in single- to double-stranded DNA conversion during conjugative plasmid transfer

**DOI:** 10.1093/nar/gkad075

**Published:** 2023-02-11

**Authors:** Minjia Shen, Kelly Goldlust, Sandra Daniel, Christian Lesterlin, Yoshiharu Yamaichi

**Affiliations:** Université Paris-Saclay, CEA, CNRS, Institute for Integrative Biology of the Cell (I2BC), 91198, Gif-sur-Yvette, France; Graduate School of Structure and Dynamics of Living Systems, Université Paris-Saclay, 91190, Gif-sur-Yvette, France; Molecular Microbiology and Structural Biochemistry (MMSB), Université Lyon 1, CNRS, Inserm, UMR5086, 69007, Lyon, France; Université Paris-Saclay, CEA, CNRS, Institute for Integrative Biology of the Cell (I2BC), 91198, Gif-sur-Yvette, France; Molecular Microbiology and Structural Biochemistry (MMSB), Université Lyon 1, CNRS, Inserm, UMR5086, 69007, Lyon, France; Université Paris-Saclay, CEA, CNRS, Institute for Integrative Biology of the Cell (I2BC), 91198, Gif-sur-Yvette, France

## Abstract

Dissemination of antibiotic resistance, a current societal challenge, is often driven by horizontal gene transfer through bacterial conjugation. During conjugative plasmid transfer, single-stranded (ss) DNA is transferred from the donor to the recipient cell. Subsequently, a complete double-stranded (ds) plasmid molecule is generated and plasmid-encoded genes are expressed, allowing successful establishment of the transconjugant cell. Such dynamics of transmission can be modulated by host- or plasmid-encoded factors, either in the donor or in the recipient cell. We applied transposon insertion sequencing to identify host-encoded factors that affect conjugative transfer frequency in *Escherichia coli*. Disruption of the recipient *uvrD* gene decreased the acquisition frequency of conjugative plasmids belonging to different incompatibility groups. Results from various UvrD mutants suggested that dsDNA binding activity and interaction with RNA polymerase are dispensable, but ATPase activity is required for successful plasmid establishment of transconjugant cells. Live-cell microscopic imaging showed that the newly transferred ssDNA within a *uvrD^−^* recipient often failed to be converted to dsDNA. Our work suggested that in addition to its role in maintaining genome integrity, UvrD is also key for the establishment of horizontally acquired plasmid DNA that drives genome diversity and evolution.

## INTRODUCTION

Securing the integrity of the genome is important during the vertical transmission of genetic information. A multitude of DNA repair pathways have evolved to treat DNA lesions created by different endogenous and exogenous factors. Different proteins, including DNA acting enzymes such as helicases, nucleases and DNA polymerases, participate in repair pathways. The UvrD helicase of *Escherichia coli*, which belongs to superfamily 1A (SF1A) helicases with 3′ → 5′ directionality (1), is involved in three pathways: methyl-directed mismatch repair (MMR), nucleotide excision repair (NER) and transcription-coupled repair (TCR). In MutLSH-dependent MMR, UvrD removes the DNA fragment containing the misincorporated nucleotide after the cleavage by the MutH endonuclease (2). For UvrABC-dependent NER, UvrD releases UvrC and the damaged oligonucleotide (3). UvrD’s role in TCR was discovered most recently, where it was shown that UvrD backtracks RNA polymerase (RNAP) that was stalled at a DNA lesion, thus allowing NER enzymes to gain access to the damage (4). Nonetheless, the core domains of UvrD harboring DNA binding and ATPase/helicase activities are essential for a ‘wrench-and-inchworm mechanism’ of DNA unwinding and strand/protein displacement functions (5). The latter is also involved in removal of proteins from single-stranded (ss) DNA, such as RecA (5–7). In addition, the ∼75 amino acid extreme carboxy-terminal domain (CTD) was shown to interact with a variety of proteins, with a particular importance during TCR for interaction with RNAP (8).

In contrast to vertical transmission, horizontal acquisition of genes allows much faster evolution of the genome. Conjugation is one of the three means of horizontal gene transfer mechanism along with transformation and transduction. In general, conjugative plasmids and chromosomally encoded integrative and conjugative elements (ICEs) are the substrate of transfer from the donor to the recipient cell. Conjugative plasmids and ICEs often encode genes to confer resistance to antibiotics, and they are responsible for 80% of acquired antibiotic resistance in bacteria (9). During conjugation, a mating pair between donor and recipient cells is established, and then ssDNA is peeled off from the plasmid origin of transfer site (*oriT*) in the donor. This reaction was carried out by a plasmid-encoded relaxase protein, which harbors nickase and helicase activities, of which the latter belongs to superfamily 1B with 5′ → 3′ unwinding polarity (1). Relaxase is then covalently bound to the transferring strand of DNA (T-strand) and translocated from the donor cell to the recipient cell via a type 4 secretion system (T4SS), presumably through the T4SS pilus lumen (10,11). Within the recipient cell, the newly acquired ssDNA plasmid is recircularized and converted into a double-stranded (ds) DNA plasmid. Subsequently, the expression of plasmid genes establishes the plasmid and converts recipient into a transconjugant cell. Eventually, a transconjugant cell can act as a new donor. This sequence of events is well documented [e.g. (12,13)]. However, due to previous technological limitations, we lacked a detailed understanding of the molecular mechanisms, particularly for the reactions occurring within the recipient cell between DNA entry and the eventual establishment of a transconjugant cell.

Conjugative plasmids encode genes essential for their transfer, including the conjugation machinery and DNA mobilization proteins. In many cases, their expression is regulated to limit the capacity of transfer to a subpopulation (12,13). Mutations in these transfer genes can nullify or modulate transfer ability, including certain mutations that can enhance transfer efficiency. Enhancing mutations further threaten the propagation of antibiotic resistance via conjugation through the creation of derepressed or superspreader mutants (14–17). For example, a naturally occurring transposon (Tn) insertion into the *tir* gene was involved in a higher transfer frequency of the plasmid pOXA-48a and its dissemination in the Enterobacteriaceae (15). Additionally, a past study highlighted the isolation of a superspreader mutant of pESBL, a plasmid associated with an enterohemorrhagic/aggregative *E. coli* outbreak in Germany in 2011 (16,17). The study of pESBL utilized a method, Tnseq, that involved Tn insertion library construction and next-generation sequencing (NGS) (18). Tnseq was also used to identify transfer genes and the plasmid maintenance loci of several conjugative plasmids (16,19,20). Here, we took a Tnseq approach to screen host-encoded factors that can modulate the efficiency of conjugative plasmid transfer. To this end, in addition to clinically relevant pESBL, we also used the classical *E. coli* F plasmid and the broad-host-range conjugative plasmid R388 as test plasmids. Our Tnseq screen involving the three different plasmids suggested the UvrD helicase as a recipient factor for successful conjugative plasmid transfer. We also observed that UvrD deficiency resulted in 10–100-fold lower conjugation frequency. Genetic and novel microscopy techniques allowed us to pinpoint the activity of UvrD involved in ssDNA to dsDNA conversion, and deduce a new mechanistic model to explain its involvement in conjugative plasmid transfer.

## MATERIALS AND METHODS

### Strains, plasmids and growth conditions

Unless otherwise specified, bacterial cells were grown in lysogeny broth (LB) liquid medium at 37°C with agitation at 180 rpm or LB solid medium with 1.5% (w/v) agar. Supplements were used at the following concentrations, when appropriate: ampicillin, 100 μg/ml; tetracycline, 10 μg/ml; chloramphenicol, 25 μg/ml; trimethoprim, 10 μg/ml; kanamycin (Km), 50 μg/ml; streptomycin (Sm), 100 μg/ml; gentamicin, 5 μg/ml; diaminopimelic acid, 300 μM; arabinose 0.02%. Cell growth curve experiments were done with a microplate reader (Tecan Infinite M200 Pro, Tecan, Switzerland), as described previously (17).

Conjugation and P1 transduction were used to move plasmids (pESBL, F, R388 and pCVD442-based allelic exchange plasmids) and chromosomal mutations between different *E. coli* backgrounds, respectively. Allelic transduction along with the antibiotic markers was confirmed when necessary by PCR. Point mutations of UvrD, including [E665*] for ΔCTD, were introduced by double crossovers with pCVD442-based plasmids (21). *In vivo* expression of Flp recombinase using pCP20 was used to remove FRT-flanked Km resistance cassettes (22). RecA overexpression plasmids were constructed in pBAD33 vector (23).

Strains, plasmids and oligonucleotides used in this study are listed in Supplementary Tables S1, S2 and S3, respectively.

### Conjugation frequency test

Transfer frequencies of conjugative plasmid were measured as described previously (17). Briefly, overnight cultures of recipient (100 μl) and donor (10 μl) cells were sedimented and resuspended in 50 μl of LB. Cell mixture was then placed on a 0.45-μm HAWP filter (Merck, Darmstadt, Germany) upon an LB agar plate. Following incubation at 37°C for 2 h, cells were resuspended within 1 ml of LB and spread onto LB plates containing relevant antibiotic(s) to count the colony forming units (CFU) of donor, recipient and transconjugant cells. Conjugation frequency is defined as the number of transconjugant cells divided by the number of donors.

### Transposon insertion sequencing

Transposon insertion libraries were created following protocols described in (20), except using MFD*pir* as the host for random delivery of transposon (24). In brief, overnight cultures of bEYY2154 (MFD*pir*/pEE18, the Tn donor) and MC1061 were mixed and incubated for 2 h. Cells with the Km^R^*Himar1* Tn inserted in the MC1061 genome were recovered on plates as Sm^R^ Km^R^ colonies (∼210 000 to cover the genome) that were scraped and collected as the ‘recipient library’. For the two independent Tnseq experiments, one batch of a recipient library was previously prepared (24) and the other was created during this study. To prepare the ‘transconjugant library’, *E. coli* cells (donor) harboring pESBL, F or R388 were mixed with ∼10^8^ recipient library cells. After 2 h of conjugation at 37°C, cell mixture was resuspended and plated to obtain colonies of transconjugant cells in the presence of relevant antibiotic selection. NGS library preparation, including gDNA preparation, MmeI digestion, adapter ligation and PCR amplification, was carried out as described in (20). Illumina sequencing was carried out at the I2BC NGS facility.

### Transposon insertion sequencing data analysis

Tnseq results were analyzed as described previously (20). Essentially, after all reads were trimmed of adaptor sequences by Cutadapt (25), the resulting ∼16 nt reads were mapped to the *E. coli* genome (MG1655 chromosome and corresponding plasmid sequence) using Bowtie (26). If a read mapped multiple times on the reference genome, it was randomly distributed to one of the sites. Mapped reads at every TA site were counted and visualized with Artemis genome browser (27) for initial inspection.

### Determination of plasmid copy number

Whole genome DNA was extracted from exponential cell culture by GenElute Bacterial Genomic DNA Kit (Merck). Approximately 10 ng of DNA was subjected to quantitative PCR using the CFX384 Real-Time System (Bio-Rad, Hercules, CA, USA) with the SYBR Green I Master reagent (Roche, Basel, Switzerland) or Luna Universal qPCR reagent (New England Biolabs, Ipswich, MA, USA). *narW* locus was used for chromosome quantification and *repZ*, *tetR* and *orf7*_R388_ were used for pESBL, F and R388 plasmid quantification, respectively. Plasmid DNA containing both the chromosomal and plasmid fragments (pEYY3, pEYY161 and pEYY413 for pESBL, F and R388, respectively) was used to draw standard curves.

### Single-nucleotide polymorphism analysis

NGS libraries of test JJC2530 and parental JJC40 gDNA were prepared by GenElute Bacterial Genomic DNA Kit and NEBNext Ultra II DNA Library Prep Kit for Illumina (New England Biolabs). Illumina sequencing (paired-end 50 bp) results were analyzed as described previously (28). Illumina sequencing was carried out at the I2BC NGS facility.

### Microscopy

For the snapshot analysis, cells were grown in rich defined media, EZ medium (hereafter referred to as EZ; Teknova, Hollister, CA, USA). Overnight cultures of recipient (100 μl) and donor (30 μl) cells were mixed and washed once with EZ. Cells were resuspended in 50 μl of EZ and deposited onto a filter membrane and upon an LB agar plate. After 2 h of incubation, the filter was resuspended in 1 ml of EZ to collect the cells, which was subsequently mixed with another 1 ml of EZ. With appropriate dilutions, cells were plated on LB agar plates containing relevant antibiotics for selecting donor, recipient and transconjugant cells. In parallel, a drop of cells was examined for snapshot microscopy analyses. One microliter of cell culture was spotted onto an agarose pad (1%, w/v), and then phase contrast and mCherry signals were acquired using a DM600-B microscope (Leica Microsystems, Wetzlar, Germany) with Prime BSI Scientific CMOS camera (Teledyne Photometrics, Tucson, AZ, USA).

For the time-lapse analysis, overnight cultures in M9-CASA (M9 medium supplemented with 0.4% casamino acid and 0.2% glucose) were diluted to an OD_600nm_ of 0.05 and grown until an OD_600nm_ of 0.8. Conjugation samples were obtained by mixing 50 μl of donor and 50 μl of recipient into an Eppendorf tube. Subsequently, 50 μl of the mix was loaded into a B04A microfluidic chamber (ONIX, CellASIC^®^) (29). Nutrient supply was maintained at 1 psi and the temperature maintained at 37°C throughout the imaging process. Cells were imaged every 1 min for 90–120 min. Time-lapse fluorescence microscopy imaging was carried out on an Eclipse Ti2-E microscope (Nikon, Tokyo, Japan), equipped with ×100/1.45 oil Plan Apo Lambda phase objective, ORCA-Fusion digital CMOS camera (Hamamatsu, Hamamatsu, Japan) and using NIS software for image acquisition. Acquisition was performed using 50% power of a Fluo LED Spectra X light source at 488 and 560 nm excitation wavelengths. Exposure settings were 100 ms for Ssb-YFP and 100 ms for mCherry-ParB_pMT1_, and 50 ms for phase contrast.

## RESULTS

### Tnseq unveiled the involvement of UvrD during conjugative plasmid transfer

To screen for host-encoded factors that modulate conjugative plasmid transfer, we utilized Tnseq experiments. We used high-density transposon insertion mutant libraries of *E. coli* as the recipient cells for the conjugative transfer of three different conjugative plasmids, pESBL, F and R388 (which belong to IncI, IncF and IncW incompatibility groups, respectively), from another *E. coli* strain as the donor. Transconjugant cells grown under selection with appropriate antibiotics were collected as the transconjugant library, and Tnseq results were compared between before and after the conjugation, i.e. recipient and transconjugant libraries. Mutations that could facilitate or disturb conjugative transfer of plasmids would show increased or decreased Tnseq reads in the transconjugant library than the recipient library.

**Figure 1.**
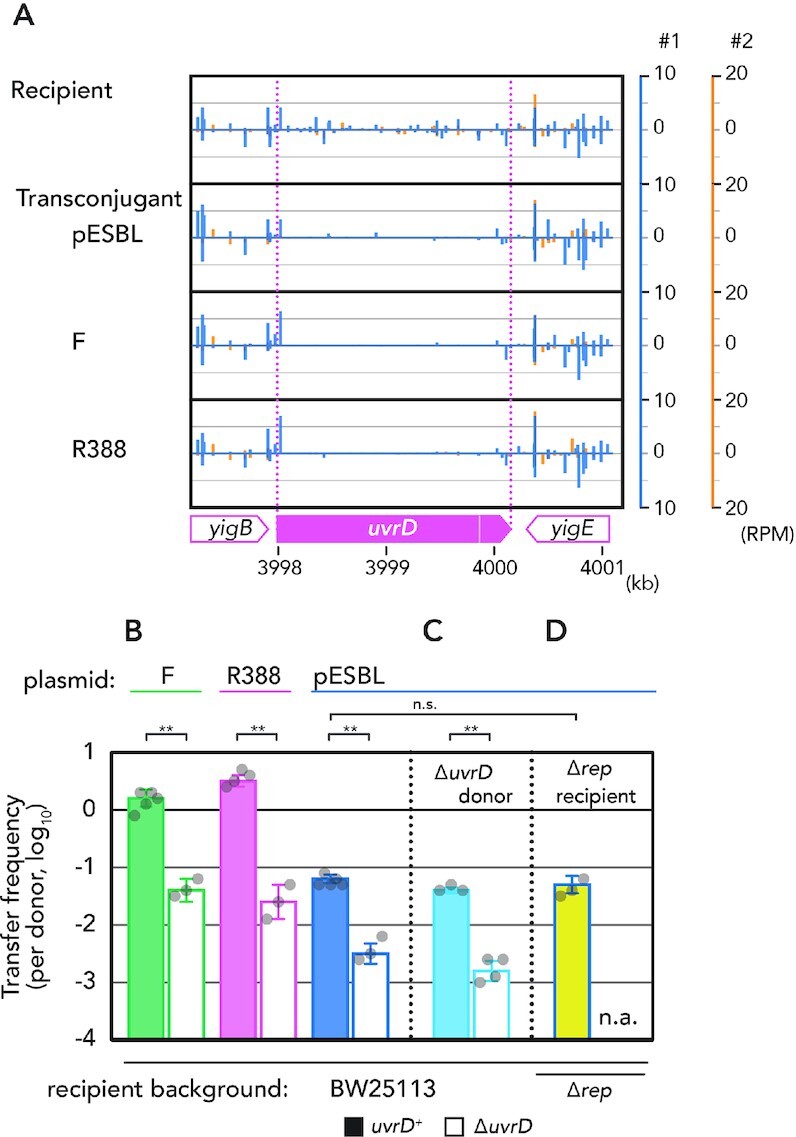
Decrease in conjugative plasmid transfer in a *uvrD* mutant recipient. (**A**) Tnseq reads mapped on the *uvrD* and its flanking region. Vertical lines correspond to fraction of reads in respect to direction of transposon. Results of two independent experiments are shown. RPM, reads per million reads mapped. (**B**–**D**) Conjugation frequency of indicated plasmid in different genetic backgrounds. The average and standard deviations along with individual data points are shown. n.a., not applicable due to synthetic lethality. *, *P* < 0.05; **, *P* < 0.01; n.s., not significant, by two-sided Student’s *t*-test.

We found that the *uvrD* gene consistently contained very few Tnseq reads in the transconjugant libraries across the three conjugative plasmids, and consistently across the two independent experiments (Figure 1A). To ascertain whether a *uvrD* defect in the recipient cell resulted in decreased plasmid transfer, we used a *uvrD* deletion mutant and tested conjugation frequency of pESBL, F and R388 plasmids. As shown in Figure 1B, all the three plasmids showed 10–100-fold lower conjugation frequency to a Δ*uvrD* recipient compared to a *uvrD*^+^ control. Note that *uvrD* mutant did not show a growth defect including viability in a conjugation frequency test (Supplementary Figure S1). *Escherichia coli* encodes another SF1A helicase, Rep. However, the Tnseq results suggested that transposon-based disruption of *rep* did not affect conjugative plasmid transfer. Subsequently, we verified that a Δ*rep* recipient strain did not affect the transfer frequency of three conjugative plasmids we tested (Figure 1D and Supplementary Figure S2A).

### The effect of *uvrD* mutation on conjugative transfer is specific to the recipient

It has been previously shown that UvrD is involved in the rolling-circle replication of certain plasmid types, by unwinding the nicked dsDNA plasmid (30,31). Thus, we examined whether the transfer frequency defect of the three conjugative plasmids in the Δ*uvrD* background is a consequence of impaired replication. Quantitative PCR results indicated comparable copy number of pESBL, F and R388 plasmids in *uvrD*^+^ and Δ*uvrD* host cells (Supplementary Figure S1C). Furthermore, a Δ*uvrD* donor strain did not affect plasmid transfer frequency (Figure 1C and Supplementary Figure S2A). These results suggest that the effect of Δ*uvrD* is specific to the recipient, presumably during the establishment of transconjugant cells. Hereafter, we focused on the involvement of recipient UvrD during conjugative plasmid transfer, mainly using pESBL as the test plasmid.

### A specific activity of the multifunctional UvrD protein is involved in plasmid transfer

**Figure 2.**
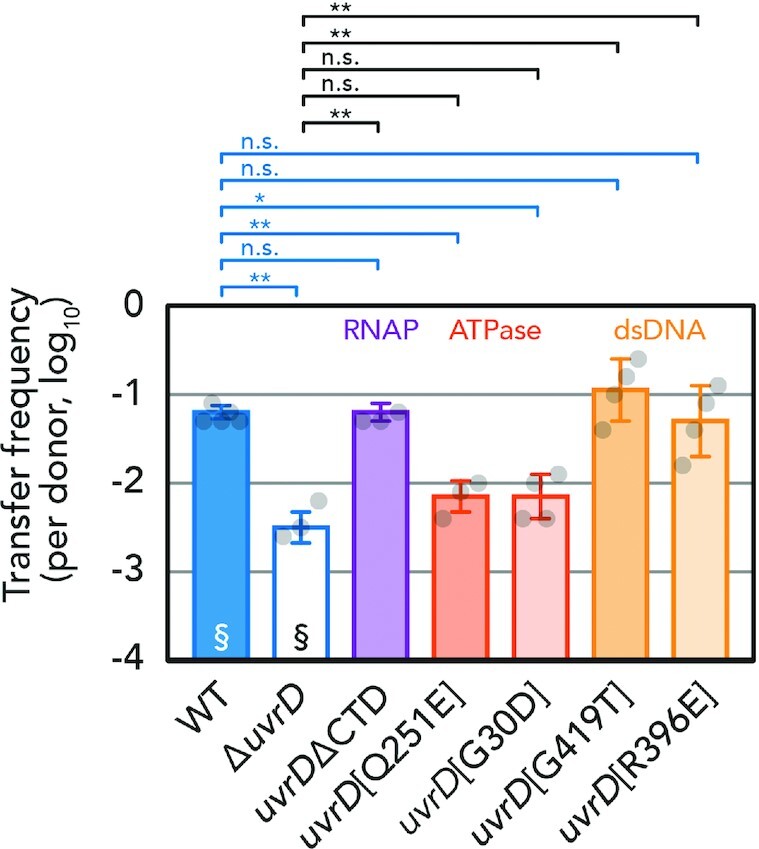
Conjugation frequency of different UvrD mutants. Recipient UvrD mutants are indicated in groups. Average and standard deviations along with individual data points are shown. §, recapitulated from Figure 1B for comparison. *, *P* < 0.05; **, *P* < 0.01; n.s., not significant, by two-sided Student’s *t*-test.

In addition to involvement in rolling-circle plasmid replication, multiple functions of UvrD concern reactions related to DNA repair. Regarding the TCR, we constructed a CTD deletion mutant (ΔCTD) of UvrD. Comparable plasmid transfer frequency was obtained between wild type (WT) and ΔCTD (Figure 2), suggesting that UvrD interaction with RNAP is not important for conjugative plasmid transfer.

A mutant of UvrD, UvrD [Q251E] harbors a mutation in a conserved helicase domain and was shown to be defective in both NER and MMR pathways (32). Furthermore, another mutant allele of *uvrD*, *uvrD252* (or formerly *recL152*), which contains a glycine to aspartic acid mutation in the ATP-binding domain (hereafter referred to as *uvrD* [G30D]), exhibits sensitivity to UV irradiation while only slightly more sensitive to exposure to an alkylating agent compared to the WT protein (33,34). The measured transfer frequency of pESBL into these two ATPase mutants ([Q251E], [G30D]) was comparable and as low as the Δ*uvrD* host (Figure 2).

A previous structure–function study characterized further mutants of UvrD. Among them, [G419T] and [R396E] mutants retained normal ATPase activity and ssDNA binding but severely reduced binding to dsDNA (5). Interestingly, a WT level of conjugation frequency was retained in these dsDNA binding mutants (Figure 2). Altogether, these results suggest that the ATPase activity of UvrD is required for its proper function in plasmid transfer, but its interactions with dsDNA and RNAP are dispensable.

### Genetic interaction with RecA suggests UvrD function during conjugation

**Figure 3.**
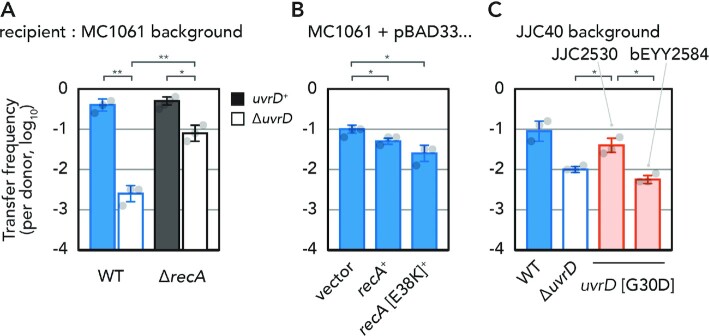
Conjugation frequency of pESBL in different recipient backgrounds. (**A**) Epistasis analysis with Δ*recA*. (**B**) Effect of RecA overexpression. (**C**) Difference of conjugation frequency with *uvrD* [G30D] (*uvrD252*) allele in the original JJC40 background. Average and standard deviations along with individual data points are shown. *, *P* < 0.05; **, *P* < 0.01, by two-sided Student’s *t*-test.

While Rep is constitutively expressed, UvrD is overexpressed during the SOS response. SOS is induced by RecA and results in the activation of various DNA recombination and repair pathways. We tested the effect of *recA* mutation on conjugative plasmid transfer. Consistent with our Tnseq results, a Δ*recA* mutation itself did not have a significant effect on conjugation frequency (Figure 3A). However, we found that the decreased conjugation frequency of pESBL into a Δ*uvrD* strain is rescued by an additional Δ*recA* mutation (Figure 3A). Furthermore, similar results were also obtained in other conjugative plasmids, F and R388 (Supplementary Figure S2B). These results indicate that RecA is epistatic to UvrD for conjugative plasmid transfer.

Regarding the relationship between UvrD and RecA, prior *in vivo* and *in vitro* results suggested that UvrD removes RecA from DNA (6,7,35). Since the conjugation frequency of Δ*recA* strain is at the WT level, a plausible scenario is that RecA–DNA interaction in the recipient cell somehow limits conjugation frequency, and UvrD can solve the problem. To test this possibility, we aimed to increase RecA–DNA interaction by overexpression of RecA, either the WT protein or a variant, RecA [E38K], which was shown to form nucleoprotein filament on DNA with greater persistence against UvrD’s stripping activity (7). When RecA was ectopically expressed in recipient *E. coli* from an overexpression vector, conjugation frequency of pESBL was decreased (Figure 3B). Notably, the decrease of conjugation frequency was more apparent with the E38K mutant RecA (Figure 3B). Similar results were also obtained for F and R388 plasmids (Supplementary Figure S2C).

Furthermore, thanks to RecA–DNA interaction, GFP-tagged RecA exhibits foci in *E. coli* cells (35). It was shown that, in the Δ*uvrD* background, the number of foci per cell and intensity of GFP signal are higher than those found in the *uvrD^+^* host. This phenotype can be suppressed by an additional mutation in *recQ* (35). Similar genetic interactions were also found in plasmid transfer. Our initial characterization of the UvrD [G30D] mutation was performed in the *uvrD252* strain we obtained, known as JJC2530. This strain exhibited an intermediate conjugation frequency between WT (*uvrD*^+^) and Δ*uvrD* strains of the corresponding JJC40 background (Figure 3C). However, when the *uvrD* [G30D] allele was introduced into a different background including BW25113 (Figure 2), or even independently re-introduced into the original JJC40 (Figure 3C, bEYY2584) strain background, the new strains showed plasmid conjugation frequency as low as the Δ*uvrD* mutant. We assumed there could be a suppressor mutation in the genome of JJC2530. Whole genome sequencing and single-nucleotide polymorphism (SNP) analysis indeed showed that JJC2530 harbors a point mutation that caused a G269E mutation in RecQ. This 269th glycine residue is highly conserved among bacterial and eukaryotic RecQ homologs (36).

Altogether, we interpret that increased RecA–DNA interaction in the recipient cell can impede successful conjugative plasmid transfer, and UvrD functions to counteract the interaction.

### Microscopic observations revealed defective dsDNA conversion during conjugation

So far, conjugation frequency was calculated by the formation of colonies on selective media, which is the ultimate output of conjugative plasmid transfer. To further understand the involvement of UvrD in conjugative plasmid transfer, we took advantage of microscopy to dissect the different steps of plasmid establishment at the cellular level.

**Figure 4.**
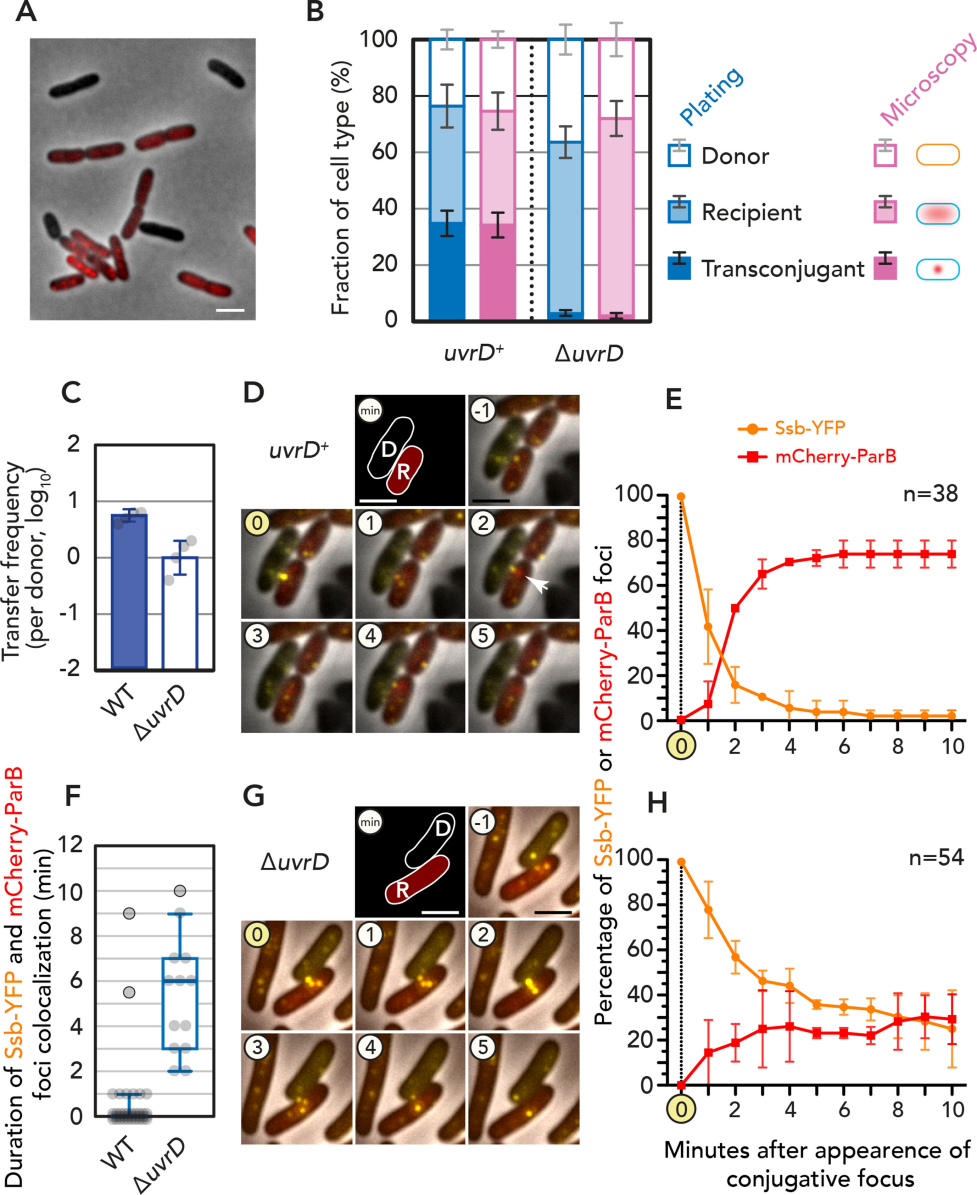
Visualization of plasmid transfer processes. (**A**) Representative field of cells containing donor (pESBL::*parS_pMT1_*), recipient (*mcherry-parB_pMT1_^+^*) and transconjugant population. Bar = 2 μm. (**B**) Comparison of fraction of cells measured by microscopy and CFU (plating). Average and standard deviations of three experiments are shown. (**C**) Conjugation frequency of pESBL Hft::Tn superspreader mutant to indicated recipient cells. Average and standard deviations along with individual data points are shown. (**D**, **G**) Representative time-lapse images of conjugation. The frame that exhibited Ssb-YFP conjugation focus was set to 0 min. Formation of mCherry-ParB _pMT1_ focus is indicated by arrow. D, donor; R, recipient. Bar = 2 μm. (**F**) Colocalization period between Ssb-YFP and mCherry-ParB_pMT1_ foci shown in Tukey’s box-and-whisker plot. Individual data points are also shown (**E**, **H**). Percentage of cells exhibiting Ssb-YFP and/or mCherry-ParB_pMT1_ foci. Average and standard deviation of two independent experiments are shown. *n* stands for the number of total conjugation events analyzed.

We utilized ParB/*parS* system for subcellular visualization of pESBL (20). In brief, the *parS* sequence from pMT1 plasmid (*parS_pMT1_*) was inserted in pESBL, and cognate ParB_pMT1_ protein was fused to mCherry and expressed from the host chromosome. A donor strain that harbors pESBL::*parS_pMT1_* but does not encode the fluorescent mCherry-ParB_pMT1_ protein is dark under microscopy. In contrast, the recipient strain expresses mCherry-ParB_pMT1_, and exhibits red diffuse signals, as it lacks the corresponding DNA binding site, *parS_pMT1_*. After a conjugative plasmid transfer event has taken place, the ds plasmid (or a partially replicated intermediate containing a ds *parS_pMT1_* site) is formed and mCherry-ParB_pMT1_ signals convert into foci (Figure 4A). As ParB does not bind to the ssDNA version of *parS* (37,38), this system acts as a sensor for the conversion of ssDNA to dsDNA during plasmid establishment in the recipient (39).

In Figure 4B, we compare the fraction of cells exhibiting mCherry-ParB_pMT1_ foci with the proportions of transconjugant cells calculated by colony formation. In the normal *uvrD*^+^ recipient, the proportions of each cell type are roughly the same by both measurement techniques. Similarly, in Δ*uvrD* recipients the decreased number of transconjugant cells measured corresponds to the lack of cells exhibiting mCherry-ParB_pMT1_ foci under microscopy. These results suggest that the involvement of UvrD in plasmid transfer acts before the ssDNA was converted into dsDNA in the recipient cell.

To further investigate the DNA actions under microscopy, we took advantage of fluorescently labeled *E. coli* single strand binding protein (Ssb-YFP). This protein has been used to visualize the entry of the F plasmid ssDNA into recipient cells, which formed bright ‘conjugative foci’ at the entry point of the plasmid in recipient cells (40). Conversion of ssDNA to dsDNA can be monitored by dual labeling of Ssb-YFP and mCherry-ParB_pMT1_, as the disappearance of Ssb-YFP foci is followed by the formation of the mCherry-ParB_pMT1_ foci (40). To facilitate the observation of pESBL conjugation events, here we took advantage of a superspreader mutant plasmid (17), and Ssb-YFP was also expressed in the donor cells. Consistent with the parental pESBL, the superspreader mutant also showed ∼10-fold lower conjugation frequency to Δ*uvrD* recipient (Figure 4C). In *uvrD*^+^ recipient cells, the formation of Ssb-YFP conjugative foci was followed by the rapid formation of mCherry-ParB_pMT1_ foci, revealing the successful conversion of ssDNA to dsDNA (Figure 4D and E). While the Δ*uvrD* recipient exhibited Ssb-YFP conjugative foci at similar frequency, ssDNA to dsDNA conversion was dramatically impaired in Δ*uvrD* recipients. This is reflected by the delayed and partial disappearance of Ssb-YFP conjugative foci, and the reduced formation of mCherry-ParB_pMT1_ foci (Figure 4G and H). Furthermore, in Δ*uvrD* recipients, colocalization of Ssb-YFP and mCherry-ParB_pMT1_ foci was sometimes observed over longer periods of time (Figure 4F), which suggests that the conversion of ss to ds plasmid DNA can often be aborted or halted. These results confirm that UvrD is important for plasmid conjugation by ensuring the successful ss to ds conversion of the newly internalized plasmids, rather than transport between the donor and recipient cells.

### UvrD function in conjugative plasmid transfer is conserved

**Figure 5.**
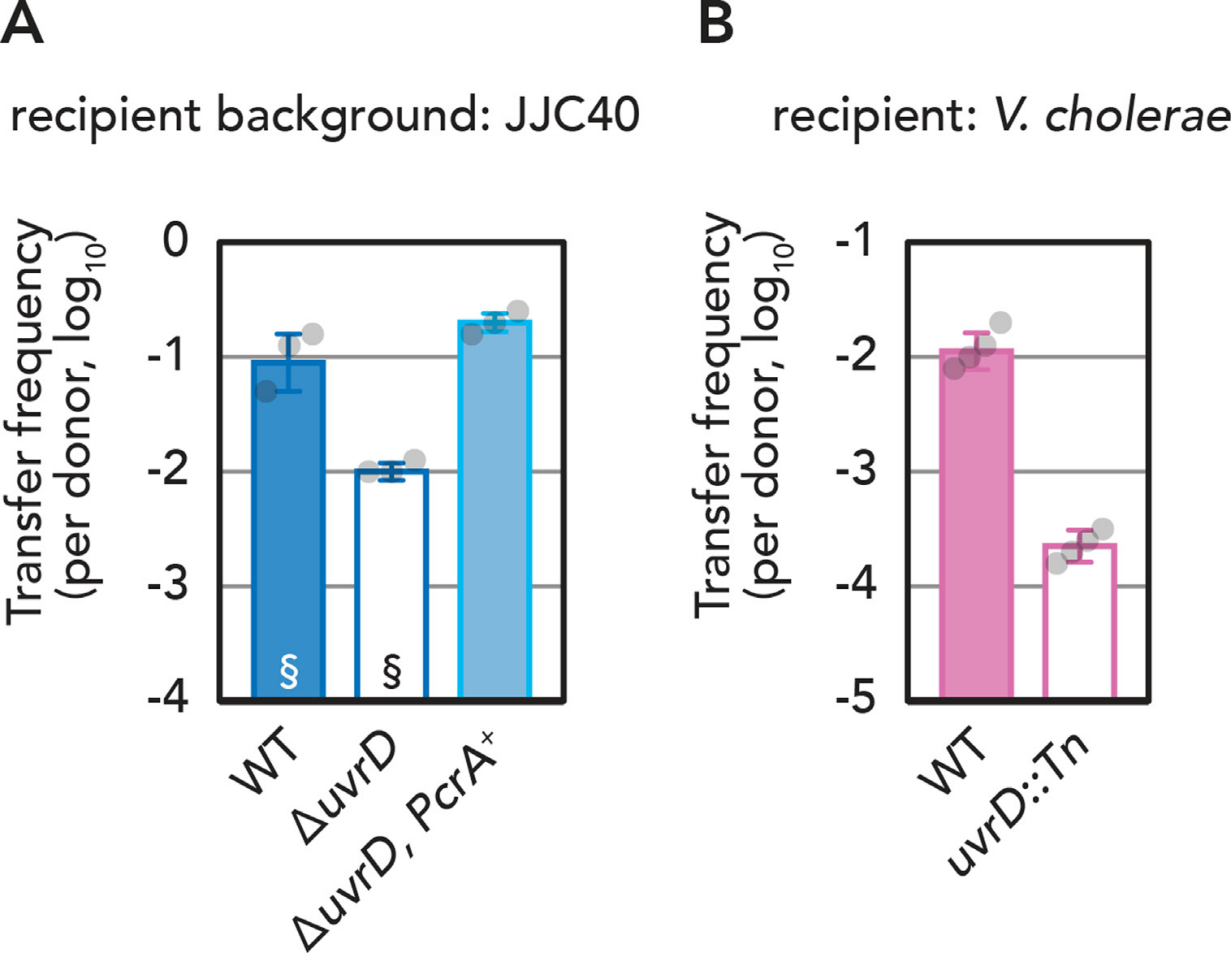
Function of UvrD homologs. (**A**) Conjugation frequency of pESBL in *E. coli* strain, where Δ*uvrD* is complemented by *B. subtilis* PcrA. §, recapitulated from Figure 2B for comparison. (**B**) Conjugation frequency of R388 from *E. coli* donor to *V. cholerae* recipient with or without *uvrD*. The average and standard deviations along with individual data points are shown.

UvrD homologs are widely conserved (41), and PcrA, a UvrD homolog encoded in Gram-positive bacteria such as *Bacillus subtilis*, is known to be able to complement the UV sensitivity of *E. coli* Δ*uvrD* (42). We tested whether PcrA can rescue the Δ*uvrD* defect in the conjugation process. As shown in Figure 5A, conjugation frequency of pESBL to the Δ*uvrD PcrA*^+^*E. coli* was comparable to the parental *uvrD^+^ E. coli*, suggesting that PcrA also has a function in conjugative plasmid transfer, presumably in conversion of ssDNA to dsDNA. Finally, we examined whether *uvrD* deficiency in other bacterial species shows similar effects on conjugative plasmid transfer. To this end, we chose *Vibrio cholerae*, which is a Gram-negative, gamma-proteobacterium but not a member of the Enterobacteriaceae. Due to the narrow host range of pESBL and F (restricted to enterobacteria), the broad-host-range IncW plasmid R388 was used in this analysis. Similarly to *E. coli* (Figure 1B), the conjugation frequency of R388 was ∼100-fold lower in a *uvrD* mutant of *V. cholerae* (Figure 5B), strongly suggesting that UvrD function during conjugation is rather conserved.

## DISCUSSION

In this study, we utilized a Tnseq-based screen in *E. coli* to investigate host-encoded factors that can modulate the transfer frequency of conjugative plasmids. To this end, three different plasmids belonging to the IncI, IncF and IncW incompatibility groups were used to identify general and/or plasmid-specific host factor(s). Even though Tnseq suggested a few genes that can modulate plasmid transfer, only UvrD was confirmed to be involved by retesting the transfer frequencies of deletion mutants. In addition, Δ*uvrD* had effect on conjugation frequency in all the three plasmids, and no plasmid-specific host factor was identified in this study.

Tnseq was previously used to search host factors involved in the conjugative transfer of an ICE in *B. subtilis*, ICE*Bs1* (43). Many of the genes identified are implicated in the cellular envelope. Considering our results, the UvrD homolog PcrA should also play a role in the conjugation of ICE*Bs1*. However, it was not identified by Tnseq, presumably because PcrA is essential for growth in *B. subtilis* (unless additional suppressor mutation such as Δ*recF*) (43,44). A high-throughput analysis to identify *E. coli* host factors involved in the transfer of R388 had also been previously carried out (45). There, a Δ*uvrD* mutation was claimed to show decreased conjugation frequency but only ∼40% from the WT level. The difference appears to be due to the donor/recipient ratio of conjugation assay. The effect of Δ*uvrD* could have been underestimated due to the donor/recipient ratio [∼1:1 in (45), compared to ∼1:10 in this study] if the conjugation frequency for the WT was saturated. Nonetheless, our genetic and microscopy results unambiguously showed the involvement of UvrD in conjugative plasmid transfer.

We showed evidence that the involvement of UvrD homologs in conjugation frequency in the recipient is likely common across many bacterial species. In contrast, the conservation of function in the donor cell is still elusive. While our results demonstrated that UvrD mutation in the donor had no effect on conjugation frequency and plasmid copy number (Figure 1C and Supplementary Figure S1C), *B. subtilis* PcrA is required for both replication and conjugation of ICE*Bs1* transfer in the donor cell (44,46).

Plasmid genes at the first region to enter the recipient cell (leading region) are usually repressed in the donor cells and expressed upon the entry of plasmid DNA in the recipient cell (13,39,47,48). A recent paper showed that translocation of proteins encoded in the leading region from the donor to the recipient cell through T4SS barely happens, even if they are overexpressed ectopically (49). However, a plasmid-encoded DNA methyltransferase (M.EcoGIX) is suggested to function in the recipient cell as the dsDNA complementation occurs (50). These findings highlight the importance of the expression of certain plasmid-encoded genes at the early stage of conjugation, even before dsDNA complementation. Indeed, the vast majority of leading region genes are encoded on the T-strand, so that the ssDNA is the template for both transcription and complementation of dsDNA plasmid. It is conceivable to assume that transcription–replication conflicts may limit successful gene expression and/or dsDNA plasmid synthesis, even though codirectional one is not as deleterious as head-on collision (51). UvrD is known to have an important role during TCR and its CTD is essential for backtracking the stalled RNAP away from the DNA lesion. In our Tnseq results, considerable Tn insertions were detected in the CTD in the transconjugant libraries (Figure 1A). Consistently, the plasmid transfer frequency of a ΔCTD mutant showed that CTD is dispensable for UvrD function during conjugative plasmid transfer (Figure 2). These results suggest that a stalled RNAP on T-strand is unlikely to be the cause of abortive plasmid establishment. Similarly, two UvrD mutants that are defective in dsDNA binding but retained normal ssDNA binding activity (5) exhibited a WT level of plasmid transfer frequency. These results suggest that a strand/protein displacement function, rather than DNA unwinding function, takes place during the establishment of a conjugative plasmid. This is reasonable as the substrate for UvrD in this situation is presumed to be ssDNA. In contrast, the ATPase activity remained essential for UvrD involvement in successful conjugation.

In addition to genetics, we utilized several microscopy reporters to monitor the key steps of the conjugation process at the cellular level. It allowed us to determine at which stage the abortion of conjugative plasmid transfer took place in the Δ*uvrD* recipient. In addition to cells taking a much longer time or even failing to exhibit the mCherry-ParB_pMT1_ ds plasmid DNA focus, Ssb-YFP conjugative foci also exhibited increased dwelling time (Figure 4E and H). These two phenotypes are consistent with the impaired ability of the conversion of ssDNA to dsDNA in the recipient cells that lack the active UvrD protein. These results suggest that either UvrD and Ssb compete on ssDNA or UvrD could strip Ssb off the T-strand. As we demonstrated that the ATPase activity of UvrD is required, the latter scenario is likely the case. It is reasonable to assume that T-strand ssDNA can also be a substrate for RecA filament formation. Indeed, it has been suggested that Ssb facilitates binding and elongation of RecA filaments on the ssDNA templates, which result in melting the secondary structures (e.g. hairpins and loops) (52,53). In the F plasmid, it was shown that hairpin formation at *Frpo* locus in the leading region allows initiation of transcription and production of RNA, which subsequently serves to prime the synthesis of complementary strand DNA (54). *In vitro*, UvrD was shown to dismantle RecA filaments formed on ssDNA and the ATPase activity is required for the disassembly function (6,7).

Altogether, we propose a model where upon entry plasmid ssDNA will be coated by Ssb and RecA. This protein coating would normally disrupt formation of hairpin structures on the ssDNA. UvrD would act on the nucleoprotein complex to dismantle RecA (6,7) and possibly Ssb as well, so that hairpins can be formed on the ss plasmid DNA, which facilitate ss to ds plasmid DNA conversion. While highly dynamic RecA filaments for homologous recombination are observed (55,56), detailed visualization of RecA during the conjugation with high spatiotemporal resolution is yet to be accomplished. However, our genetic results, including the epistasis of Δ*recA* with Δ*uvrD*, as well as effect of overexpression of a RecA mutant that is less prone to be removed from ssDNA by UvrD, fit with our model.

In summary, we have shown yet another function of the highly versatile UvrD helicase. We implicate UvrD in a crucial role for ssDNA to dsDNA conversion during the establishment of conjugative plasmids. While UvrD has established roles in DNA repair in the pursuit of genome integrity, the new function we have revealed drives the genetic diversity and evolution that is inherent to the accumulation of plasmid DNA.

## DATA AVAILABILITY

Illumina sequencing results for Tnseq and SNP analysis are available at ArrayExpress (https://www.ebi.ac.uk/arrayexpress/) with accession numbers E-MTAB-11704 and E-MTAB-11704, respectively.

Other data supporting the findings of this study are available within the article, including in the Supplementary Data.

## Supplementary Material

gkad075_Supplemental_Files
