# Supplementary material for: Recipient UvrD helicase is involved in single- to double-stranded DNA conversion during conjugative plasmid transfer
Source: Nucleic Acids Res. 2023 Feb 11;51(6):2790–9. doi: 10.1093/nar/gkad075 (PMC10085688; doi:10.1093/nar/gkad075)
Supplement: gkad075_Supplemental_Files [file gkad075_supplemental_files.zip › gkad075 SupData_proof_ref_all.pdf]

SUPPLEMENTARY MATERIALS FOR

Recipient UvrD helicase is involved in single- to double-stranded DNA conversion during the conjugative plasmid transfer

Minjia Shen, Kelly Goldlust, Sandra Daniel, Christian Lesterlin, Yoshiharu Yamaichi

Legend to Supplementary Figures

Figure S1. Growth rate, fitness and plasmid copy number in Δ*uvrD* cells. (A) Growth rate of *E. coli* cells in the absence (LB) or presence of indicated anitibiotics. Individual data points, average and standard deviations of 3 experiments are shown. (B) Individual colony forming units (CFUs) of conjugation efficiency experiments shown in Figure 1B. Results of mock experiment which $uvrD^{+}$ *and* Δ*uvrD* recipient cells were incubated without donor cells are also presented. (C) Copy number of plasmids in $uvrD^{+}$ and Δ*uvrD* host. The average and standard deviations along with individual data points were shown.

Figure S2. Conjugation frequencies of F and R388 plasmids. (A) Conjugation frequency with Δ*uvrD* donor and Δ*rep* recipient, complementing results of Figure 1B. §, recapitulated form Figure 1B for comparison. (B) Epistasis analysis with Δ*recA*, complementing results of Figure 3A. Average and standard deviations along with individual data points were shown. (C) Effect of RecA overexpression, complementing results of Figure 3B. *, $p<0.05$; **, $p<0.01$, by two-sided Student's t-test.

Supplementary Table S1. Strains used in this study

Strain	Description/Genotype	Reference
Escherichia coli		
DH5α λ*pir*	Host strain for the cloning of R6K*ori* plasmid	Laboratory stock
SM10 λ*pir*	Host strain to deliver allelic exchange plasmids	Laboratory stock
MFD*pir*	Host strain to deliver the Tn for Tnseq	(1)
MC1061	*hsdR2 hsdM$^+$ hsdS$^+$ araD139 Δ(ara-leu)7697 Δ(lac)X74 galE15 galK16 rpsL mcrA mcrB1*	Laboratory stock
BW25113	*F- DE(araD-araB)567 lacZ4787(del)::rrnB-3 LAM- rph-1 DE(rhaD-rhaB)568 hsdR514*	(2)
MKW278	MG1655 Δ*lacZ::cat*	(3)
bEYY1097	MC1061 / pESBL Hft::TnΔkan	(4)
bEYY1666	MC1061 / F1-10	Laboratory stock
bEYY2003	MKW278 / F1-10	This study
bEYY2072	MC1061 / pESBL *IG_hp23::parS$_{pMT1}$*	(5)
bEYY2118	BW25113 Δ*galK::mcherry-parB$_{pMT1}$_kan*	(5)
bEYY2139	MC1061 / pESBL Hft::TnΔkan *IG_hp23::parS$_{pMT1}$*	This study
bEYY2154	MFD*pir* / pEE18	(6)
bEYY2181	MC1061 / R388	Laboratory stock
bEYY2229	MG1655 ori::*tetO* array::*gent* / pESBL	This study
bEYY2234	BW25113 Δ*galK::mcherry-parB$_{pMT1}$*	This study
bEYY2310	MG1655 ori::*tetO* array::*gent* / R388	This study
bEYY2311	MKW278 / R388	This study
bEYY2361	MC1061 Δ*uvrD::kan*	This study
bEYY2389	MC1061 Δ*recA::kan*	This study
bEYY2392	MC1061 Δ*uvrD*	This study
bEYY2411	MC1061 Δ*uvrD* Δ*recA::kan*	This study
bEYY2458	BW25113 Δ*galK::mcherry-parB$_{pMT1}$* Δ*uvrD::kan*	This study
bEYY2460	MC1061 Δ*uvrD* / pESBL	This study
bEYY2546	BW25113 Δ*galK::mcherry-parB$_{pMT1}$* *uvrD*ΔCTD	This study
bEYY2547	BW25113 Δ*galK::mcherry-parB$_{pMT1}$* *uvrD*ΔCTD *rpsL*	This study
bEYY2573	BW25113 Δ*galK::mcherry-parB$_{pMT1}$* *uvrD* [G30D]	This study
bEYY2574	BW25113 Δ*galK::mcherry-parB$_{pMT1}$* *uvrD* [Q251E]	This study
bEYY2575	BW25113 Δ*galK::mcherry-parB$_{pMT1}$* *uvrD* [Q251E] *rpsL*	This study
bEYY2578	BW25113 Δ*galK::mcherry-parB$_{pMT1}$* *uvrD* [G30D] *rpsL*	This study
bEYY2584	JJC40 *sfiA11 uvrD* [G30D]	This study
bEYY2585	BW25113 *ssb-yfp_kan*	This study
bEYY2587	BW25113 *uvrD* [G419T]	This study
bEYY2591	BW25113 Δ*uvrD ssb-yfp_kan*	This study
bEYY2592	BW25113 *uvrD* [R396E]	This study
bEYY2594	BW25113 *uvrD* [G419T] *rpsL*	This study
bEYY2596	BW25113 *uvrD* [R396E] *rpsL*	This study
bEYY2654	MC1061 Δ*uvrD* / R388	This study
bEYY2655	MC1061 Δ*uvrD* / F	This study
bEYY2661	BW25113 Δ*galK::mcherry-parB$_{pMT1}$_kan* / pESBL	This study
bEYY2662	MC1061 Δ*uvrD* Δ*recA::kan* / pEYY493	This study
bEYY2663	MC1061 Δ*uvrD* Δ*recA::kan* / pBAD33	This study
bEYY2664	MC1061 / pBAD33	This study
bEYY2665	MC1061 / pEYY493	This study
bEYY2667	MC1061 / pEYY496	This study
bEYY2670	MC1061 Δ*uvrD::kan* / pBAD33	This study
bEYY2672	MC1061 Δ*uvrD::kan* / pEYY493	This study

bEYY2674	MG1655 ori::*tetO* array::*gent* / F	This study
IL05	AB1157 ori::*tetO* array::*gent* ter::*lacO* array::*kan*	(7)
JJC40	*thi-1 proA2 argE3 lacY1 galK2 ara-14 xyl-15 mtl-1 tsx-33 rpsL31 supE44 hsdR* Thr$^+$ Pro$^+$	(8)
JJC2530	JJC40 *sfiA11 uvrD252* (*recQ* [G269E]) *	(8)
JJC2642	JJC40 *sfiA11 ΔuvrD-294::kan*	(8)
JJC2673	JJC40 *sfiA11 ΔuvrD-294::kan lacZ::pcrA*$^+$	(8)
JW3784	BW25113 Δ*uvrD::kan*	(9)
JW5604	BW25113 Δ*rep::kan*	(3)
LY117	MG1655 *rpsL ssb-yfp_kan*	Laboratory stock
LY2332	MG1655 *rpsL ssb-yfp_kan* / pESBL Hft::TnΔkan *IG_hp23::*$parS_{pMT1}$	This study
LY2333	BW25113 *ssb-yfp_kan* / pSN70	This study
LY2334	BW25113 Δ*uvrD ssb-yfp_kan* / pSN70	This study
YBB1192	MKW278 / pESBL	(3)
YBB1195	MC1061 / pESBL	(9)
Vibrio cholerae		
C6706	*V. cholerae* O1 El Tor, *hapR*$^+$, SmR	(10)
bEYY2557	C6706 *lac*$^-$ *uvrD::TnFGL3*	(10)

* see main text

Supplementary Table S2. Plasmids used in this study

Plasmid	Description	Construction / Reference
pBAD33	p15A$_{ori}$ P_{BAD} *cat*	(11)
pBluescript II	*bla*	Laboratory stock
pCP20	*rep*$_{ts}$ *bla cat FRT*$^{+}$	(2)
pCVD442	*R6K*$_{ori}$ *mobRP4 bla sacB*	(12)
pEYY3	pBluescript II *narW repZ*	(3)
pEYY261	pBluescript II *narW tetR*	3 pieces Gibson assembly: *narW* fragment amplified with oYo1 x oYo608, *tetR* fragment amplified with oYo609 x oYo610, and pBluescript II / SacI + KpnI
pEYY413	pBluescript II *narW repZ orf7*$_{R388}$	2 pieces Gibson assembly: *orf7*$_{R388}$ fragment amplified with oYo1134 x oYo1117 and pEYY3 backbone amplified with oYo1177 x oYo1178
pEYY468	pCVD442 for *uvrD* [ΔCTD]	3 pieces Gibson assembly: left arm amplified with oYo1405 x oYo1391, right arm amplified with oYo1392 x oYo1406, and pCVD442 / SmaI
pEYY479	pCVD442 for *uvrD[Q251E]*	3 pieces Gibson assembly: left arm amplified with oYo1405 x oYo1438, right arm amplified with oYo1437 x oYo1406, and pCVD442 / SmaI
pEYY480	pCVD442 for *uvrD* [G30D]	2 pieces Gibson assembly: *uvrD252* and flanking region amplified with oYo1405 x oYo1406, and pCVD442 / SmaI
pEYY482	pCVD442 for *uvrD* [G419T]	3 pieces Gibson assembly: left arm amplified with oYo1405 x oYo1443, right arm amplified with oYo1442 x oYo1406, and pCVD442 / SmaI
pEYY483	pCVD442 for *uvrD* [R396E]	3 pieces Gibson assembly: left arm amplified with oYo1405 x oYo1445, right arm amplified with oYo1444 x oYo1406, and pCVD442 / SmaI
pEYY493	pBAD33 *recA*	2 pieces Gibson assembly: *recA* amplified with oYo1494 x oYo1495, and pBAD33 / SacI + XbaI
pEYY496	pBAD33 *recA* [E38K]	3 pieces Gibson assembly: 5' piece amplified with oYo1495 x oYo1498, 3' piece amplified with oYo1494 x oYo1499, and pBAD33 / SacI + XbaI
pSN70	*mcherry-parB*$_{pMT1}$	(13)

Supplementary Table S3. Oligonucleotides used in this study

Name	Sequence (5'-3')
oYo1	TGTAAAACGACGGCCAGTACCTGTTCCTCTTCCCA
oYo3	TTCTTTCGCCTGATCGTCAG
oYo4	ATGAAAAGGTCGGCCTGCA
oYo7	TCATTCGGGAAGGTGTTCTG
oYo8	GGTGTTTCAGTGCATCCTGG
oYo608	AACAGGAGACAAGTGCTTAGGAGCTGTTGTGGGAATGC
oYo609	TTGCATTCCCACAACAGCTCCTAAGCACTTGTCTCCTGT
oYo610	AATTAACCCTCACTAAAGGATGAATAGTTCGACAAAGATCG
oYo611	TGGATGGAATAGCATGATGG
oYo612	AGTGGCTATTCTTCCTGC
oYo1117	ATGCAAGTCCGAGTCATCG
oYo1134	TCAATTTCGGCCATGTCG
oYo1173	GGCCGCATTCTCGATGAG
oYo1174	GCCCGCAAGAGCATGAAG
oYo1177	GACATGGCCGAAATTGAGGTCATAGCTGTTTCCT
oYo1178	TGACTCGGACTTGCATGCAGGCAGTTTCTTCAGG
oYo1391	CAACGTTACACCGACTCCTCTAGATTAGACCATCGGCGTACCCA
oYo1392	TGGGTACGCCGATGGTCTAATCTAGAGGAGTCGGTGTAACGTTG
oYo1405	CCGCATGCGATATCGAGCTCTCCCTTCGATTGAACAAGCGATGC
oYo1406	CGGATAACAATTTGTGGAATTCCCATCTCGCTTCAGGTGAAGG
oYo1437	GATCGTCGGTGATGACGACGAGTCAATCTACGGCTGGCG
oYo1438	CGCCAGCCGTAGATTGACTCGTCGTCATCACCGACGATC
oYo1442	GAATACGCCAACGCGGGGTATTACTGACCGGACGCTGGACG
oYo1443	CGTCCAGCGTCCGGTCAGTAATACCCCGCGTTGGCGTATTC
oYo1444	CAAAGATGCGCTCTCGTATCTGGAACTGATTGCCAACCGCAACG
oYo1445	CGTTGCGGTTGGCAATCAGTTCCAGATACGAGAGCGCATCTTTG
oYo1494	GCCTGCAGGTCGACTCTAGATTAAAAATCTTCGTTAGTTTCTG
oYo1495	TGGGCTAGCGAATTCGAGCTATGGCTATCGACGAAAACAAAC
oYo1498	GCGAACCGGTAGAGATGGTTTTCACATCCATGGAACGGTCTTCAC
oYo1499	GTGAAGACCGTTCCATGGATGTGAAAACCATCTCTACCGGTTCGC

REFERENCES

1. Ferrières,L., Hémery,G., Nham,T., Guérout,A.-M., Mazel,D., Beloin,C. and Ghigo,J.-M. (2010) Silent mischief: bacteriophage Mu insertions contaminate products of *Escherichia coli* random mutagenesis performed using suicidal transposon delivery plasmids mobilized by broad-host-range RP4 conjugative machinery. *J Bacteriol*, **192**, 6418–27.
2. Datsenko,K.A. and Wanner,B.L. (2000) One-step inactivation of chromosomal genes in *Escherichia coli* K-12 using PCR products. *Proc Natl Acad Sci U S A*, **97**, 6640–5.
3. Yamaichi,Y., Chao,M.C., Sasabe,J., Clark,L., Davis,B.M., Yamamoto,N., Mori,H., Kurokawa,K. and Waldor,M.K. (2015) High-resolution genetic analysis of the requirements for horizontal transmission of the ESBL plasmid from *Escherichia coli* O104:H4. *Nucleic Acids Res*, **43**, 348–60.
4. Poidevin,M., Sato,M., Altinoglu,I., Delaplace,M., Sato,C. and Yamaichi,Y. (2018) Mutation in ESBL Plasmid from *Escherichia coli* O104:H4 Leads Autoagglutination and Enhanced Plasmid Dissemination. *Front Microbiol*, **9**, 130.
5. Daniel,S., Goldlust,K., Quebre,V., Shen,M., Lesterlin,C., Bouet,J.-Y. and Yamaichi,Y. (2020) Vertical and Horizontal Transmission of ESBL Plasmid from *Escherichia coli* O104:H4. *Genes (Basel)*, **11**.
6. Espinosa,E., Yamaichi,Y. and Barre,F.-X. (2020) Protocol for High-Throughput Analysis of Sister-Chromatids Contacts. *STAR Protoc*, **1**, 100202.
7. Lau,I.F., Filipe,S.R., Søballe,B., Økstad,O.-A., Barre,F.-X. and Sherratt,D.J. (2003) Spatial and temporal organization of replicating *Escherichia coli* chromosomes. *Mol Microbiol*, **49**, 731–43.
8. Lestini,R. and Michel,B. (2007) UvrD controls the access of recombination proteins to blocked replication forks. *EMBO J*, **26**, 3804–14.
9. Baba,T., Ara,T., Hasegawa,M., Takai,Y., Okumura,Y., Baba,M., Datsenko,K.A., Tomita,M., Wanner,B.L. and Mori,H. (2006) Construction of *Escherichia coli* K-12 in-frame, single-gene knockout mutants: the Keio collection. *Mol Syst Biol*, **2**, 2006.0008.
10. Cameron,D.E., Urbach,J.M. and Mekalanos,J.J. (2008) A defined transposon mutant library and its use in identifying motility genes in *Vibrio cholerae*. *Proc Natl Acad Sci U S A*, **105**, 8736–41.
11. Guzman,L.M., Belin,D., Carson,M.J. and Beckwith,J. (1995) Tight regulation, modulation, and high-level expression by vectors containing the arabinose PBAD promoter. *J Bacteriol*, **177**, 4121–30.
12. Donnenberg,M.S. and Kaper,J.B. (1991) Construction of an *eae* deletion mutant of enteropathogenic *Escherichia coli* by using a positive-selection suicide vector. *Infect Immun*, **59**, 4310–7.
13. Nolivos,S., Cayron,J., Dedieu,A., Page,A., Delolme,F. and Lesterlin,C. (2019) Role of AcrAB-TolC multidrug efflux pump in drug-resistance acquisition by plasmid transfer. *Science*, **364**, 778–782.

Figure S1

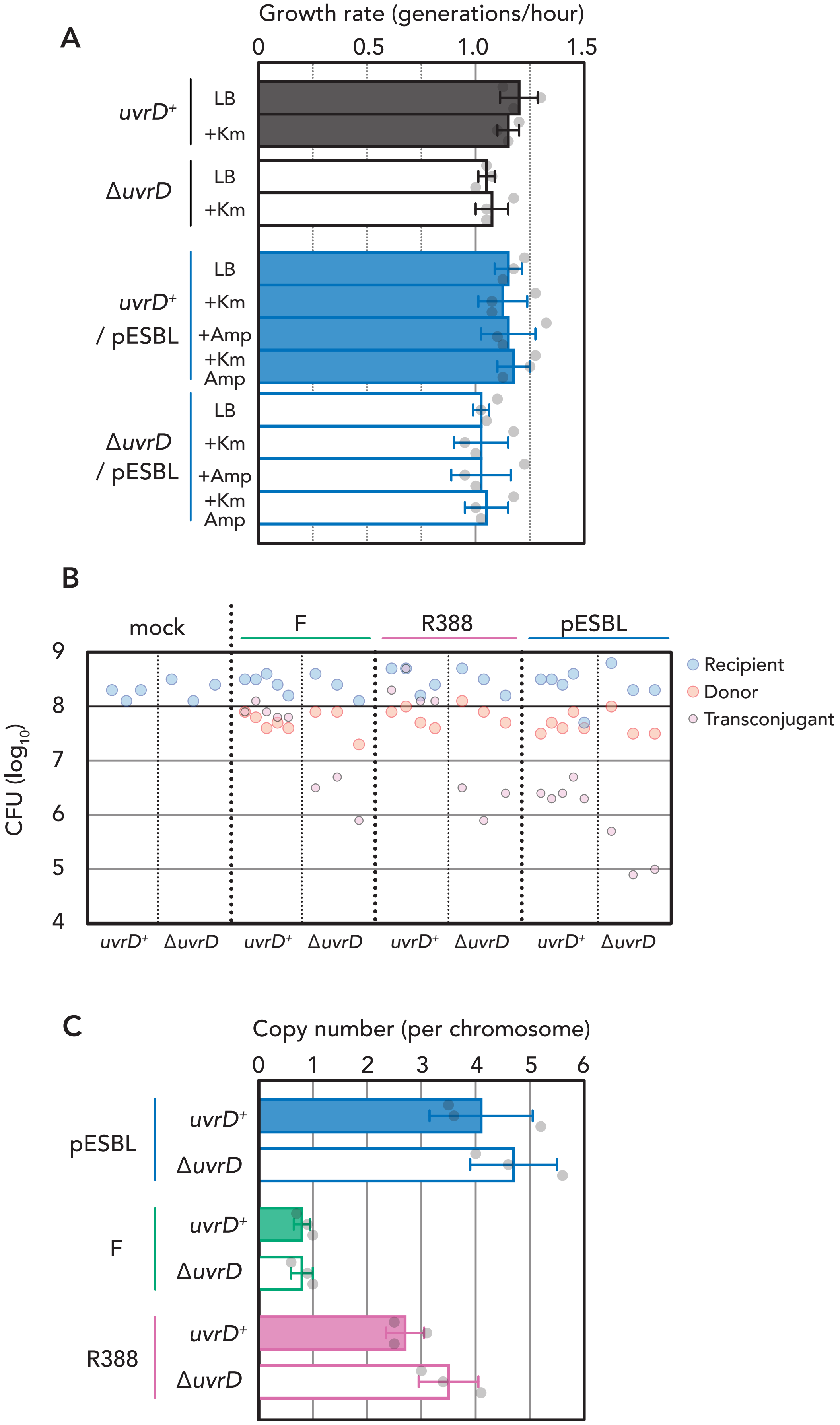

A
Growth rate (generations/hour)
0
0.5
1.0
1.5
uvrD+
LB
+Km
ΔuvrD
LB
+Km
uvrD+ / pESBL
LB
+Km
+Amp
+Km Amp
ΔuvrD / pESBL
LB
+Km
+Amp
+Km Amp
B
mock
F
R388
pESBL
CFU (log10)
9
8
7
6
5
4
uvrD+
ΔuvrD
uvrD+
ΔuvrD
uvrD+
ΔuvrD
uvrD+
ΔuvrD
Recipient
Donor
Transconjugant
C
Copy number (per chromosome)
0
1
2
3
4
5
6
pESBL
uvrD+
ΔuvrD
F
uvrD+
ΔuvrD
R388
uvrD+
ΔuvrD

Figure S2

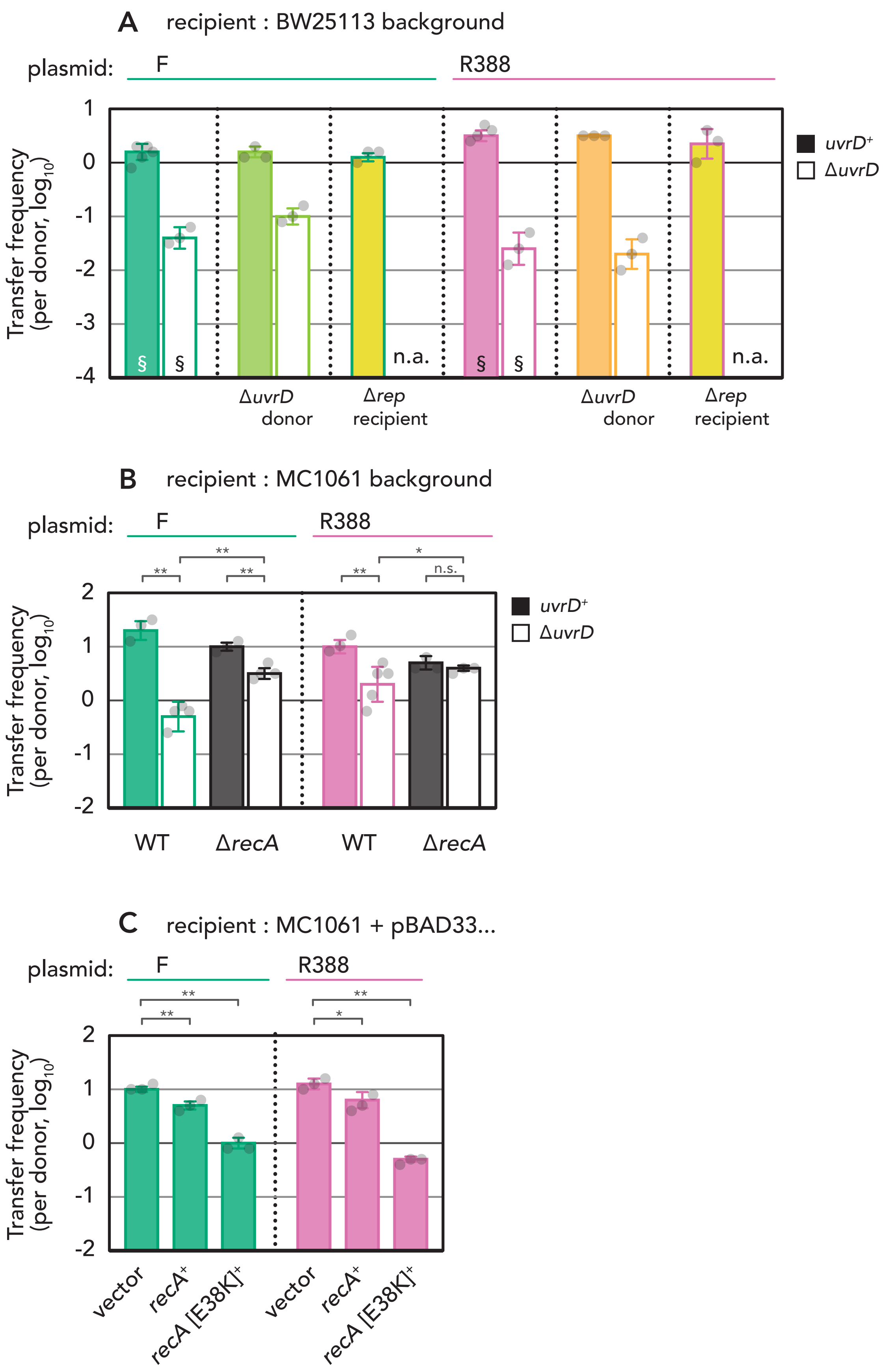

A
recipient : BW25113 background
plasmid:
F
R388
Transfer frequency (per donor, log10)
1
0
-1
-2
-3
-4
§
§
n.a.
§
§
n.a.
ΔuvrD donor
Δrep recipient
ΔuvrD donor
Δrep recipient
uvrD+
ΔuvrD
B
recipient : MC1061 background
plasmid:
F
R388
**
**
**
**
*
n.s.
2
1
0
-1
-2
WT
ΔrecA
WT
ΔrecA
uvrD+
ΔuvrD
C
recipient : MC1061 + pBAD33...
plasmid:
F
R388
**
**
**
*
vector
recA+
recA [E38K]+
vector
recA+
recA [E38K]+
